# Characterization and Rapid Gene-Mapping of Leaf Lesion Mimic Phenotype of *spl-1* Mutant in Soybean (*Glycine max* (L.) Merr.)

**DOI:** 10.3390/ijms20092193

**Published:** 2019-05-03

**Authors:** G M Al Amin, Keke Kong, Ripa Akter Sharmin, Jiejie Kong, Javaid Akhter Bhat, Tuanjie Zhao

**Affiliations:** 1 National Center for Soybean Improvement, Key Laboratory of Biology and Genetics and Breeding for Soybean, Ministry of Agriculture, State Key Laboratory of Crop Genetics and Germplasm Enhancement, Nanjing Agricultural University, Nanjing 210095, China; alamin25@gmail.com (G.M.A.A.); 2017201030@njau.edu.cn (K.K.); ripa.sharmin@gmail.com (R.A.S.); 2012094@njau.edu.cn (J.K.); 2 Department of Botany, Jagannath University, Dhaka 1100, Bangladesh

**Keywords:** soybean, spotted leaf mutant, physio-chemical performance, MutMap mapping, candidate gene

## Abstract

In plants, lesion mimic mutants (LMMs) reveal spontaneous disease-like lesions in the absence of pathogen that constitutes powerful genetic material to unravel genes underlying programmed cell death (PCD), particularly the hypersensitive response (HR). However, only a few LMMs are reported in soybean, and no related gene has been cloned until now. In the present study, we isolated a new LMM named spotted leaf-1 (*spl-1*) from NN1138-2 cultivar through ethyl methanesulfonate (EMS) treatment. The present study revealed that lesion formation might result from PCD and excessive reactive oxygen species (ROS) accumulation. The chlorophyll content was significantly reduced but antioxidant activities, viz., superoxide dismutase (SOD), peroxidase (POD) and catalase (CAT), as well as the malondialdehyde (MDA) contents, were detected higher in *spl-1* than in the wild-type. According to segregation analysis of mutant phenotype in two genetic populations, viz., W82×*spl-1* and PI378692×*spl-1*, the spotted leaf phenotype of *spl-1* is controlled by a single recessive gene named *lm1*. The *lm1* locus governing mutant phenotype of *spl-1* was first identified in 3.15 Mb genomic region on chromosome 04 through MutMap analysis, which was further verified and fine mapped by simple sequence repeat (SSR) marker-based genetic mapping. Genetic linkage analysis narrowed the genomic region (*lm1* locus) for mutant phenotype to a physical distance of ~76.23 kb. By searching against the Phytozome database, eight annotated candidate genes were found within the *lm1* region. qRT-PCR expression analysis revealed that, among these eight genes, only *Glyma.04g242300* showed highly significant expression levels in wild-type relative to the *spl-1* mutant. However, sequencing data of the CDS region showed no nucleotide difference between *spl-1* and its wild type within the coding regions of these genes but might be in the non-coding regions such as 5′ or 3′ UTR. Hence, the data of the present study are in favor of *Glyma.04g242300* being the possible candidate genes regulating the mutant phenotype of *spl-1*. However, further validation is needed to prove this function of the gene as well as its role in PCD, which in turn would be helpful to understand the mechanism and pathways involved in HR disease resistance of soybean.

## 1. Introduction

Plants have evolved complicated signaling pathways and defense system for protecting themselves against pathogen attack. Among them, hypersensitive response (HR) is the most efficient and prominent response characterized by the rapid death of plants cells that come in direct contact or are close to a pathogen. Extensive efforts have been made to identify the signaling pathway as well as to identify candidate genes involved in the control and execution of the hypersensitive cell death [1,2,3,4]. Isolation and identification of mutants in which cell death is misregulated are one of the approaches used for this study. These mutants are named as lesion mimic mutants (LMMs) showing either unregulated or constitutive cell death formation that mimic the pathogen-inducible, HR cell death [5]. Previously, LMMs have been characterized and extensively analyzed in many plant species for their responses to different plant hormones as well as modes of inheritances including groundnut [6], maize [7], *Arabidopsis thaliana* [8], rice [9,10] and barley [11,12]. However, the different pathways engaged for initiation and developments of the lesion or molecular mechanisms involved in lesion mimic, as well as basic function of the wild-type allele at a mutant locus are not well defined. Initiation and propagation of lesion on leaves of LMM plants are regulated with the age of plants, i.e., developmentally regulated [13]. Generally, the lesions first appear in the older leaves and then progress to young upper leaves [14]. Hence, LMMs are very promising genetic materials for exploring the regulatory mechanisms of plant growth and defense response.

The genes related to lesion mimic are reported to have diverse functions including a transcription factor regulating membrane receptors, superoxide dismutase, salicylate and sphingolipid signaling [15]. Genes underlying lesion mimic phenotypes appear to play direct roles in the maintenance of cellular homeostasis. Some of the lesion mimic mutant genes that have been cloned plays important role in programmed cell death (PCD) such as Ca^2+^ ion influx (*dnd1*, *dnd2*/*hlm1*, *cpr22*7 and *cpn1*/*bon1*), sphingolipid metabolism (*acd5* and *acd11*), ROS formation/sensing (*lsd1*), and porphyrin/chlorophyll biosynthesis and catabolism (*acd1*, *acd2*, *lin2*, *les22* and *flu1*) [16]. However, few LMMs, e.g., *lsd1*, accelerate the PCD with the HR-inducing bacteria, and some show normal growth, e.g., *acd5* and *cpr22* [16]. Hence, LMMs are an important tool for identifying and characterizing genes that are directly or indirectly associated with the regulation and execution of PCD in crop plants.

In soybean, T363 was the first LMM mutant to be characterized and named as *dlm* (disease-lesion mimic) [14]. The mutation in *dlm* results in the formation of small necrotic spots surrounded by chlorotic halos on leaves and is controlled by single recessive gene according to segregation ratios of the LMM trait in genetic populations [14]. Subsequently, the *dlm* phenotype was found to be light-dependent and associated with chloroplast function [15]. Inheritance of *dlm* mutant phenotype and some leaf morphological traits were carried out, and it was reported the *dlm* allele inherited independently with that of *P1*, *y9*, *f*, *lf2* and *ti* alleles controlling glabrous, chlorophyll-deficient leaf, stem trait, seven leaflet and trypsin inhibitors traits in soybean, respectively [17,18]. Wang et al. [19] found a new LMM in soybean with rugose leaf phenotype controlled by two duplicated genes, *rf1*, and *rf2*, which were mapped on chromosome 18 and 08, respectively. Although few LMMs have been characterized in soybean, the genes underlying the mutant phenotype have not been cloned yet. In addition, very little information is available about the phenomic characteristics, molecular mechanism of LMMs in soybean as well as how genes regulate PCD in soybean. In this regard, efforts are required to identify genes governing the mutant phenotype of LMMs in soybean, and to understand the molecular mechanism regulating the HR and PCD in soybean.

Map-based cloning method has been successfully implemented to identify agronomically important QTLs/candidate genes in various crops, such as wheat [20] and rice [21,22]. However, this approach has limitations being laborious, low-throughput and time-consuming in specific crops such as soybean [23]. In this regard, BSA-based whole genome re-sequencing (WGRS) approaches such as MutMap and QTL-seq methods permits rapid isolation of the genes or genomic locus responsible for the causative mutation of the phenotypes, and have been confirmed to be promising gene mapping approaches in crop plants [24,25]. Using this approach candidate genes has been successfully detected for important phenotypic characters in different crops, such as chickpea [26], barley [27], maize [28], soybean [23,29], tomato [30] and cucumber [31]. Hence, this application of WGRS in detecting the causative genes underlying mutant phenotype of crop traits will be of considerable significance in this challenging time of global hunger and the alarming global population increase [25].

In the present study, we isolated an LMM named spotted leaf-1 (*spl-1*) from the progeny of an elite soybean cultivar NN1138-2 treated with ethyl methanesulfonate (EMS), and investigated its detailed morphological and physiological characters, and then used the combined strategy of MutMap and map-based cloning mapping approaches to identify the candidate genes underlying the lesion mimic mutant phenotype of *spl-1*. The *lm1* locus controlling the *spl-1* phenotype was mapped in a 76.23 kb genomic regions on chromosome 04 harboring eight candidate genes, of which *Glyma.04g242300* was screened out as the possible target genes for *spl-1* mutant phenotype based on the qRT-PCR analysis. Hence, our findings provide new gene resources, and functional analysis of these genes will help to understand the pathway mechanism of lesion mimic as well as how plants can develop an innate immune response named hypersensitive response (HR) and programmed cell death (PCD) defense in the whole life of plants mainly from biotic and abiotic constrained.

## 2. Results

### 2.1. Phenotypic Characterization of spl-1 Mutant

**Figure 1:**
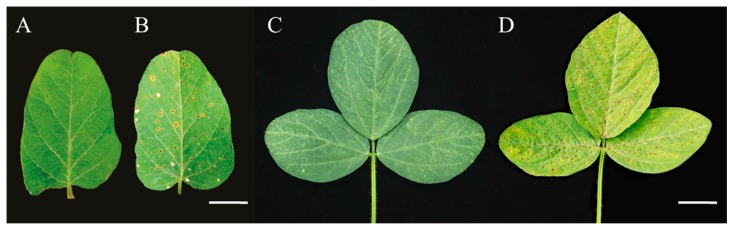
Morphological characteristics of wild-type and *spl-1* mutant soybean genotypes: (**A**,**C**) leaf phenotype characteristic of wild-type; and (**B**,**D**) leaf phenotype characteristic of *spl-1* mutant plants. Scale bars: (**A**,**B**) 1.0 cm; and (**C**,**D**) 1.5 cm.

Under natural field condition and environment, the typical tiny brown spot lesions first appeared on the lower leaves (older-leaf) of *spl-1* mutant plants after 2–3 weeks of sowing, i.e., trifoliate stage, and then progressively lesions formed on every leaf up of the plant body when the plant began to flower. The characteristic phenotype of the mutant at maturity stage were the older leaves revealed highly brown necrotic lesions of chlorotic leaves in the absence of pathogens, and early senescence was apparent, whole mutant leaves became tan, and, eventually, some died at a mature stage, unlike the wild-type. The number and size of the spots continued to increase as the leaf grew older and covered the entire leaf surface (Figure 1A–D). These results suggest that lesions on the *spl-1* mutant were developmentally regulated. The mutant phenotype was also observed to be environmentally-sensitive; the appearance of mutant phenotype was more prominent under summer-planting condition compared to spring-planting condition. Similar to our findings, Kim et al. [15] also reported that phenotype of the soybean disease-lesion mimic (*dlm1*) mutant is dependent on the light intensity, temperature, relative humidity and day length for affecting the development of cell death phenotype.

### 2.2. Leaf Pigment Content and Histological Analysis

**Figure 2:**
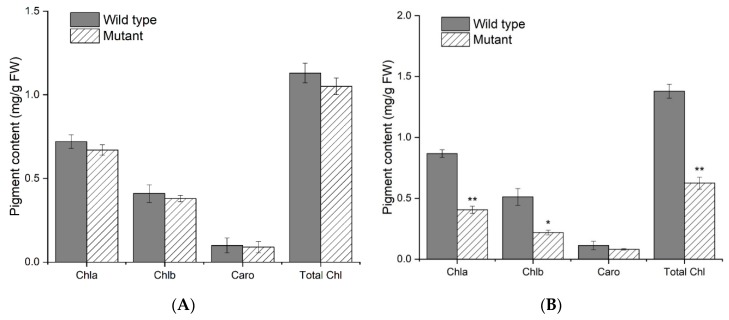
Comparison of leaf photosynthetic pigment contents in wild-type and *spl-1* mutant plants at: seedling stage (**A**); and mature stage (**B**). Chla, chlorophyll a; Chlb, chlorophyll b; Caro, carotenoids; Total Chl, total chlorophyll; FW, Fresh weight. The error bars indicate the mean ± SE (*n* = 3). SPSS software was used for statistical analysis. * significantly different at *p* < 0.05; ** significantly different at *p* < 0.01.

Chlorophyll degradation is an integral part and indicator for the degree of leaf senescence. In this study, contents of chlorophyll a, chlorophyll b, carotenoids, and total chlorophylls showed no significant difference between *spl-1* mutant and wild-type plants at the seedling stage (Figure 2A). In contrast, at maturity stage *spl-1* mutant plants showed a considerable reduction in the contents of photosynthetic pigments, with chlorophyll a, chlorophyll b and total chlorophyll decreased by 52%, 56%, and 54.6%, respectively (Figure 2B). However, no significant differences for carotenoids were found between *spl-1* mutant and wild-type at maturity stage. This result suggests that pigment accumulation in leaves was largely influenced by lesion formation in *spl-1* soybean mutant.

**Figure 3:**
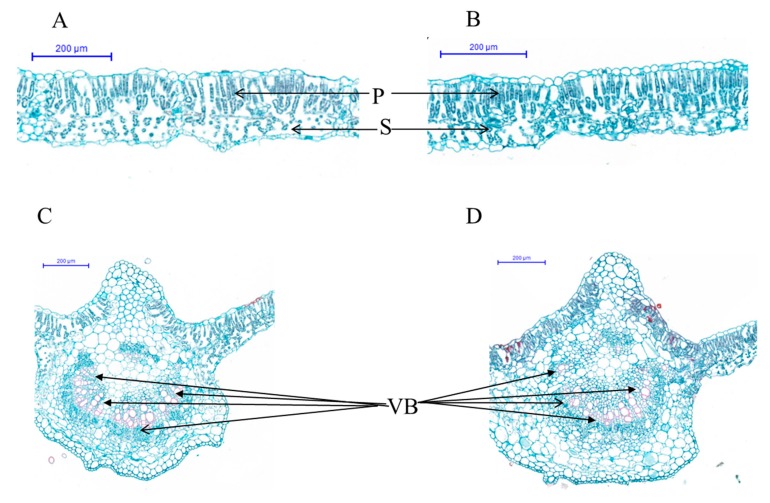
Leaf anatomical structure of wild-type and *spl-1* mutant soybean genotypes: (**A**,**C**) leaf anatomical structure of wild-type plant; and (**B**,**D**) leaf anatomical structure of *spl-1* mutant plants. *p*, palisade parenchyma; S, spongy parenchyma; VB, vascular bundle. Arrows show linear arrangement of VB in wild-type (**C**) and non-linear/distorted arrangement of VB in *spl-1* mutant (**D**).

To elucidate the leaf anatomical differences between mutant and wild-type, transverse sections of leaves from both soybean genotypes were used for histological observation (Figure 3A–D). Leaf photosynthetic mesophyll cells contain chloroplasts and are usually arranged in palisade and spongy parenchyma. In wild-type soybean, the arrangement of mesophyll cells was normal and uniform, they were well separated from each other, and both the spongy and palisade parenchyma were loosely expanded (Figure 3A). In contrast, the arrangement of the palisade and spongy parenchyma cells was highly disoriented and compact in mutant soybean (Figure 3B). In addition, our results reveal a poorly developed vascular bundle in a mutant plant, compared to normal plant that was discordant with the mesophyll expansion of mutant (Figure 3C,D). Stomata play a vital role in the gaseous exchange between leaf and outside atmosphere due to the presence of air space among the mesophyll parenchyma, which is essential for normal leaf photosynthesis [32]. Hence, compactly arranged leaf mesophyll restricted the gaseous exchanges, which in turn reduced the leaf photosynthesis resulting in chlorophyll degradation and early leaf senescence. Kura-Hotta et al. [33] reported that inactivation of photosynthesis is closely related to loss of reaction center complexes during leaf senescence of rice seedlings because the leaf hydraulic conductance (*K*_leaf_) is strictly determined by leaf venation/vascular bundle that has a strong influence on the degree to which the stomata may remain open for photosynthesis without desiccating the leaf [34]. Within and across species, *K*_leaf_ correlates strongly with stomatal pore area per leaf area, stomatal conductance and light-saturated photosynthetic rate per leaf area [34,35,36,37]. It suggests that distorted leaf anatomy might lead to reduced photosynthesis, chlorophyll degradation, lesion mimic phenotype and early leaf senescence of soybean *spl-1* mutant.

### 2.3. Physiochemical Analysis for PCD, H_2_O_2_ Accumulation and Antioxidants

**Figure 4:**
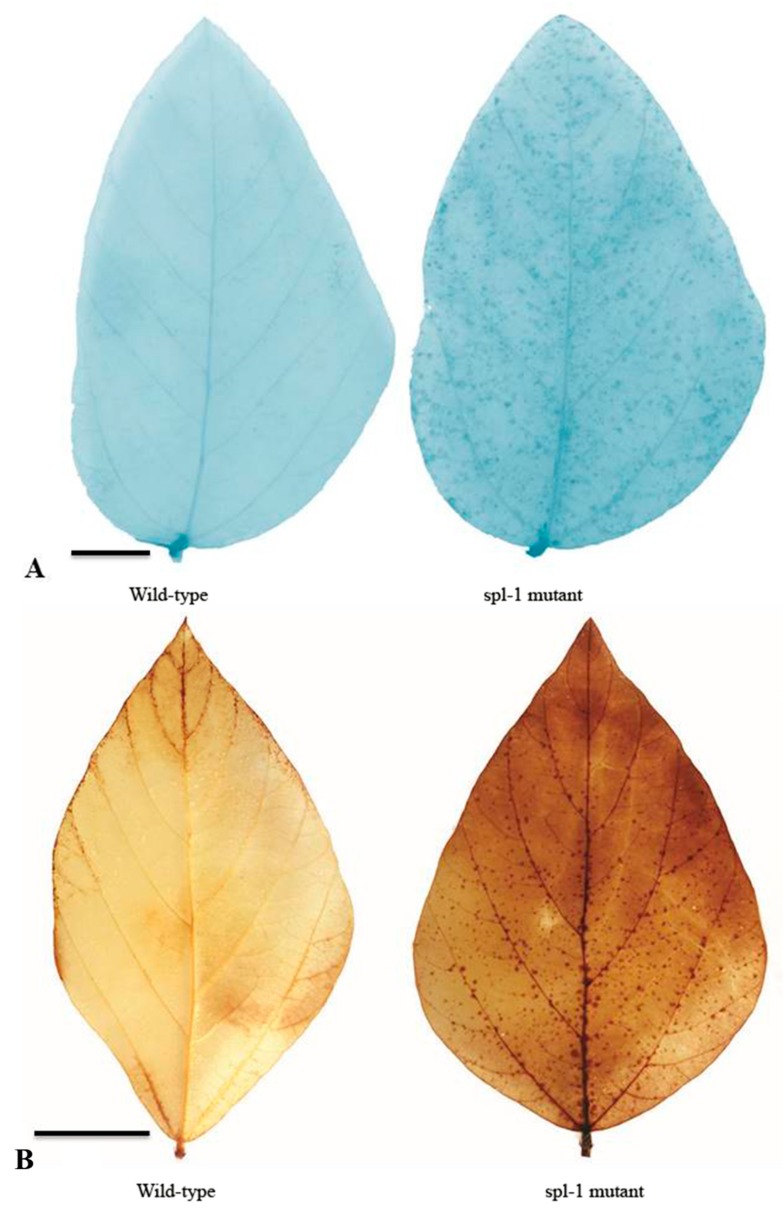
Histochemical staining analysis for leaves of wild-type and *spl-1* mutant soybean genotypes: (**A**) Trypan blue staining for cell death. The spots indicated the ROS accumulated area in the *spl-1* mutant. (**B**) DAB staining for H_2_O_2_ accumulation. Scale bars: (**A**,**B**) 1 cm.

Necrotic lesion formation usually results from PCD and ROS accumulation [38]. To determine cell death and ROS accumulation, we performed traditional methods of Trypan blue and Diaminobenzidine (DAB) staining assays, respectively [5]. After staining with Trypan blue, the leaves of *spl-1* mutant showed deep blue spots at the site of lesions, whereas the surrounding normal cells of *spl-1* mutant, as well as the whole leaf of wild-type plant, exhibited negative staining. Trypan blue staining (Figure 4A) suggested that PCD occurred during lesion formation in the *spl-1* mutant. To confirm that ROS accumulation was accompanied by PCD, we performed a DAB staining assay to assess H_2_O_2_ accumulation. After DAB staining, the leaves of *spl-1* mutant exhibited many reddish-brown spots only at necrotic sites, and dark brownish staining appeared with increasing severity of necrosis (Figure 4B), indicating a high level of H_2_O_2_ accumulation in the *spl-1* mutant. This result indicates that ROS accumulation in cells might be responsible for cell death and lesion formation, and the staining assay confirmed that the *spl-1* mutant suffered from a hypersensitive reaction and exhibited PCD with a visible phenotype at necrotic sites.

**Figure 5:**
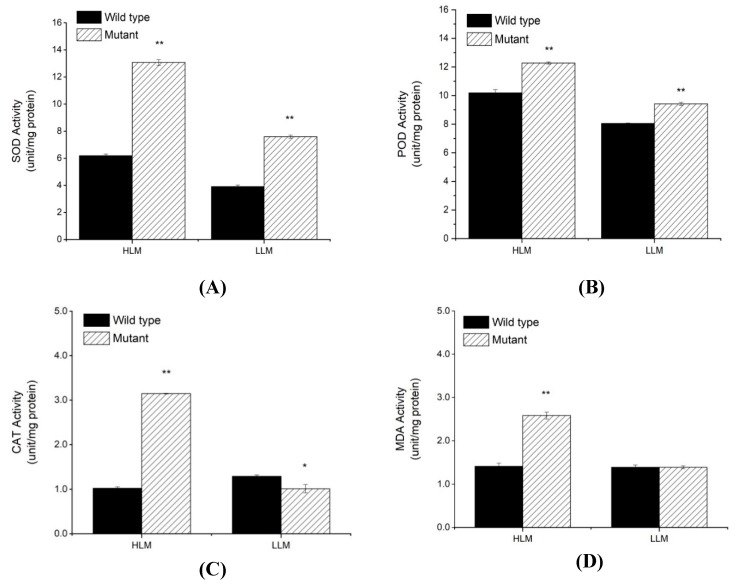
Graph showing different physiological characteristics/parameters determined for both the wild-type and *spl-1* mutant plants: (**A**) activity of superoxide dismutase (SOD); (**B**) activity of peroxidase (POD); (**C**) activity of Catalase (CAT); and (**D**) content of the malondialdehyde (MDA). The upper second leaves (lower lesion mimic (LLM)) and upper third leaves (higher lesion mimic (HLM)) of plants were used the estimation of these parameters at six weeks after sowing in pots. The data represent the means ± SE of three replicates. * significantly different at *p* < 0.05; ** significantly different at *p* < 0.01.

For further insights, we also examined some physiological changes in wild-type and mutant genotype, and evaluated the activities of some key enzymatic antioxidants, viz., SOD, POD and CAT, and also estimated the lipid peroxide content (Malondialdehyde (MDA)) at different level of lesion appearances (high lesion mimic (HLM) and low lesion mimic (LLM)) in mutant plants (Figure 5A–D). Our results show that activities of SOD, POD, and CAT were significantly higher in the *spl-1* mutant than in wild-type plants for both HLM and LLM, except for CAT that exhibited significantly lower activity at LLM in the *spl-1* mutant (Figure 5C). Furthermore, MDA content was significantly higher in *spl-1* mutant than in wild-type for HLM but revealed no significant difference at LLM. These results reveal the increased accumulation of ROS and lipid peroxides as well as the activities of antioxidants, and suggest that mutation in *spl-1* plants resulted in oxidative stress that in turn led to PCD and brown necrotic lesions on the leaf surface [39].

### 2.4. Inheritance for Spotted Leaf Trait of spl-1 Mutant

The inheritance of mutant phenotype was determined by evaluating the presence and absence of brown necrotic lesions on the leaves of F_2_ and F_2:3_ populations that were derived from the two different crosses, viz., W82×*spl-1* and PI 378692×*spl-1* (Table 1). Genetic analysis of the segregated populations revealed that F_2_ populations of W82×*spl-1* (310 plants showed wild-type phenotype and 90 plants exhibited the *spl-1* phenotype, χ^2^ = 1.20 ˂ χ^2^_0.05_ = 3.84, *p* = 0.27) and PI378692×*spl-1* (609 wild-type and 184 *spl-1* mutant, χ^2^ = 1.27 ˂ χ^2^_0.05_ = 3.84, *p* = 0.26) crosses fitted an expected 3:1 segregation ratio of wild-type to mutant. In the F_2:3_ populations of both crosses, viz., W82×*spl-1* and PI378692×*spl-1*, wild-type non-segregating and segregating lines fit 1:2 ratio (Table 1), suggesting that mutant phenotype is controlled by a single nuclear recessive gene, which is designated as *lm1*.

### 2.5. MutMap Analysis for Identification of lm1 Locus

For accelerating the mapping and identification of the genomic region for target traits, the combined strategy of WGRS and traditional map-based cloning approach with BSA have been performed. Based on mutant phenotypic data evaluation of F_2_ population derived from W82×*spl-1* cross, 20 F_2_ individuals each for wild-type and mutant were selected, and their DNA was bulked to constitute DNA Pool A (wild-type) and Pool B (mutant) for the WGRS/sequencing. After filtering, 19.36 Gb of clean data were obtained with average Q20 of 99.59% and Q30 of 98.0%, indicating the high quality of the sequencing data (Table S1). A total of 94,700,266 and 93,193,018 short reads (150 bp in length) were obtained for Pool A (96.87% coverage) and Pool B (96.96% coverage), respectively. These short reads of both pools were aligned with references genome of Williams 82, and the match rates were 95.96 and 95.84% respectively (Table S1).

**Figure 6:**
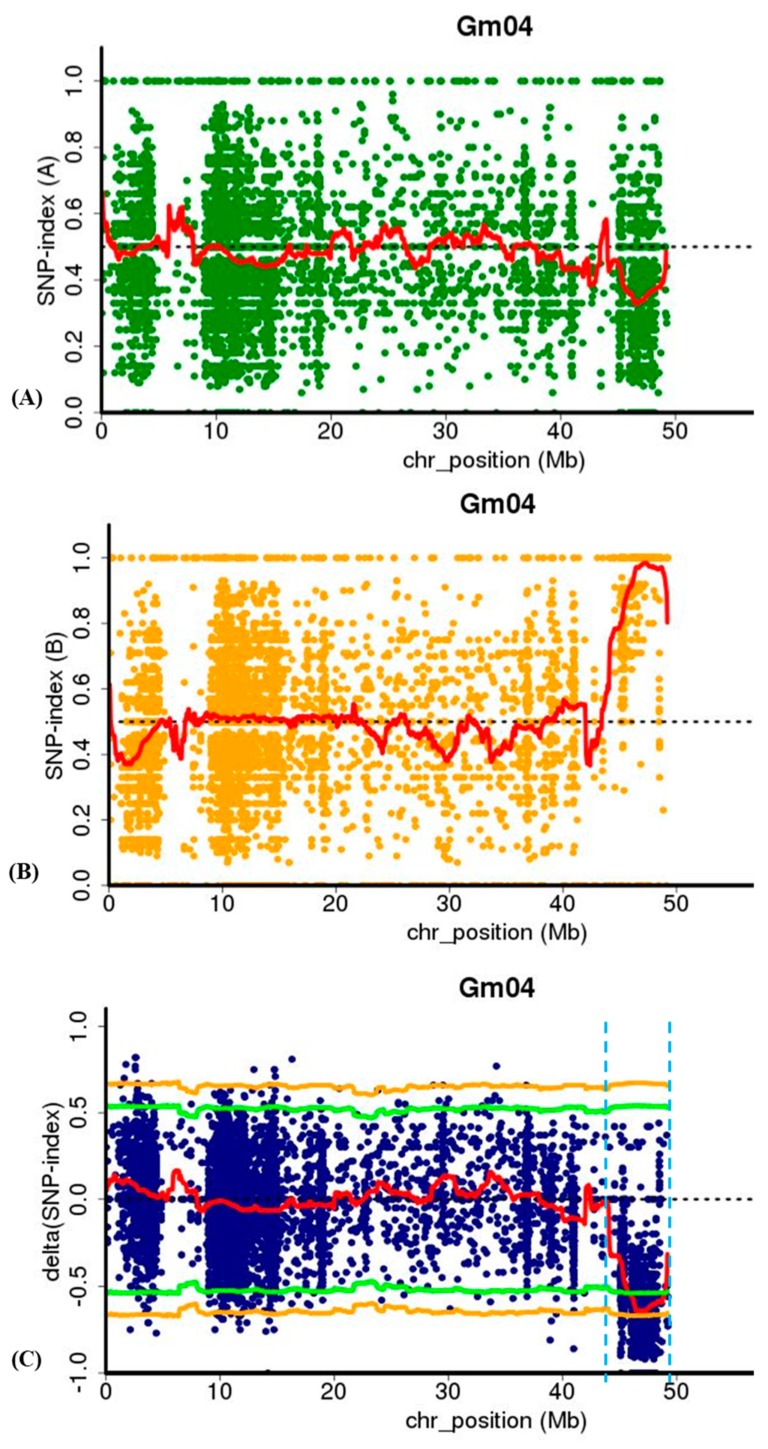
Identification of candidate genomic region (*lm1* locus) through MutMap analysis at a genomic interval of 45.80–48.95 Mb (Version Glyma v1.a1) on chromosome 04 of soybean: (**A**,**B**) the SNP-index of wild-type (A-Pool) and *spl-1* mutant (B-Pool) pools, respectively, for chromosome 04; and (**C**) the ∆ (SNP-index) plot for chromosome 04. x-axis indicates the physical position of chromosome and y-axis indicates the average SNP-index in a 2-Mb interval with a 50-kb sliding window. The Δ (SNP-index) graph was plotted with statistical confidence intervals under the null hypothesis of no QTL (*p* < 0.05). The candidate region (*lm1* locus) identified for *spl-1* mutant phenotype is marked by two red dash border lines in ∆ (SNP-index) plot.

To identify candidate genomic region associated with the mutant phenotype, SNP-index of each SNP locus in Pool A and Pool B was calculated using high-quality SNPs, those with quality score ≥ 100 and read depth ≥ 10. The average SNP-index in Pool A and Pool B and Δ (SNP-index) between Pool A and Pool B across a 2-Mb genomic interval were measured using a 50-kb sliding window and plotted for all 20 chromosomes of the soybean genome (Figure S1). Test of significance (Fisher’s exact test) was also conducted at each SNP locus for Pool A and Pool B, and the average *p*-values were calculated for SNPs located in each sliding window. In the SNP-index plotting of Pool A and Pool B, many peaks were identified. The SNP index plotting for 20 chromosomes of both the wild-type and mutant pools are provided in Figure S1, but statistical significant (*p*-value > 0.05) of only one major peak was identified in Δ (SNP-index) association analysis and were assigned as the candidate region of the gene controlling leaf lesion mimic mutant phenotype in *spl-1* mutant (Figure 6A–C and Table S2). This candidate region covers the genomic physical distance of 3.15 Mb on chromosome 04 between 45.84 and 48.95 Mb (version *Glycine max*, Wm82. a1.v1), and has Δ (SNP index) value significantly different from 0. These results indicate that a major genomic region governing lesion mimic mutant phenotype of *spl-1* was at the 3.15 Mb region of chromosome 04.

### 2.6. Validation and Fine Mapping of the lm1 Locus

To validate and narrow the candidate genomic region, i.e., *lm1* locus identified by MutMap method, we initially conducted preliminary mapping analysis by using 90 mutant plants from the F_2_ population of W82×*spl-1* cross. Out of the total 90 SSR markers in the target genomic region detected by MutMap, 18 SSR markers exhibited polymorphism between W82 and *spl-1*. Linkage analysis of segregation data by MapMaker 3.0 software revealed that the *lm1* gene was primarily located between the markers BARCSOYSSR_04_1385 and BARCSOYSSR_04_1435 in a physical distance of 655.8 kb region on chromosome 04 lying correctly in the same candidate region as identified through MutMap, and hence confirmed the results of MutMap.

**Figure 7:**
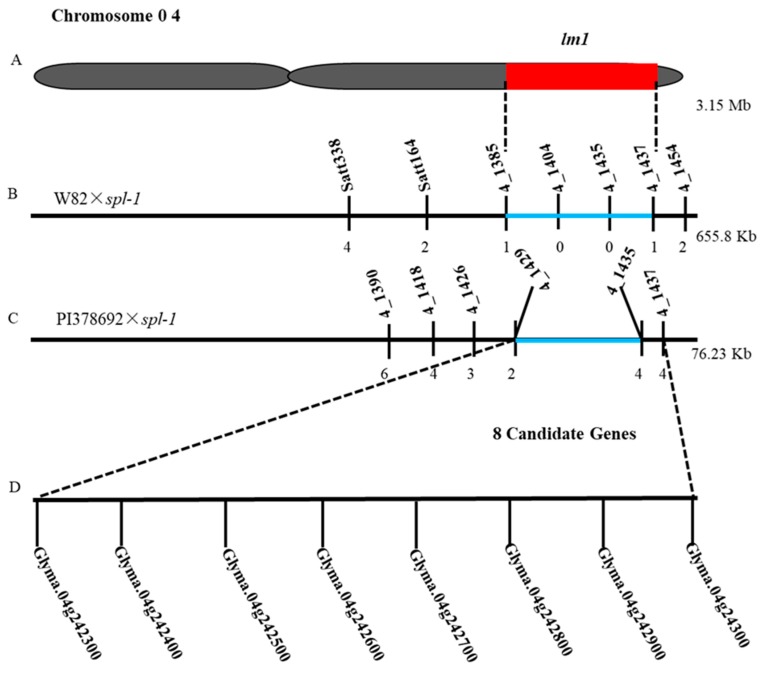
Mapping and fine mapping of *lm1* locus. (**A**) Location of *lm1* locus identified by MutMap-based BSA method on chromosome 04. (**B**) Dashes line indicated rough mapping of *spl-1* locus from cross of W82×*spl-1*. Vertical lines indicate polymorphic markers. Names of markers are shown above the line and the recombinants between *lm1* and each marker are shown below the line. (**C**) Fine mapping of *lm1* with genotyping data from newly developed polymorphic markers in the cross of PI378692×*spl-1*. (**D**) Eight candidate genes in the fine-mapped region.

The above genomic region detected through preliminary mapping was further fine-mapped by using 197 F_2_ and F_2:3_ *spl-1* mutant lines from the cross of PI378692×*spl-1*. By selecting randomly 40 pairs of SSR markers within the chromosome region identified through preliminary mapping, seven were polymorphic between PI378692 and *spl-1* and were used for further analysis (Table 2). Using genetic linkage analysis, the *lm1* gene was positioned between BARCSOYSSR_04_1429 and BARCSOYSSR_04_1435 markers, covering the physical distance of ~76.23 kb (Figure 7). By considering the reference genome sequence of Williams 82 [40] (Version Glyma 2.0), eight candidate genes were present in the genomic region of the *lm1* locus, which was narrowed to a 76.23 kb interval by fine mapping (Table 3). These genes include *Glyma.04g242300*, *Glyma.04g242400*, *Glyma.04g242500*, *Glyma.04g242600*, *Glyma.04g242700*, *Glyma.04g242800*, *Glyma.04g242900*, and *Glyma.04g243000* (Table 3). Among these eight genes, the functional annotation of six genes are known, whereas it is not available for two genes, viz., *Glyma.04g242400* and *Glyma.04g242600*, in public database (Table 3). Based on the function, *Glyma.04g242300* was considered as the probable candidate being a member of plantacyanin gene family, which belongs to sub-family of blue copper proteins, which functions in the electron transport chain during photosynthesis [41]. To further clarify it, we subject all eight candidate genes to qRT-PCR expression analysis, as discussed below.

### 2.7. qRT-PCR Expression and Sequences Analysis of Candidate Genes

**Figure 8:**
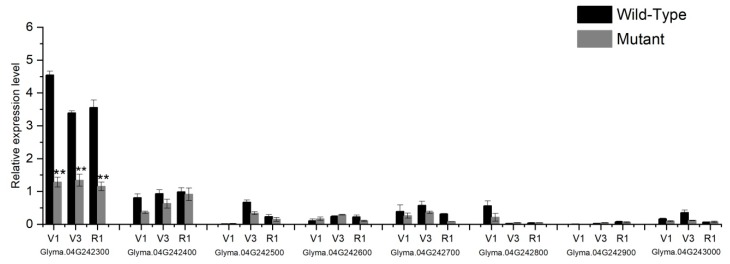
Relative gene expression of eight candidate genes in the leaves of wild-type and mutant (*spl-1*) plants at three developmental growth stages V1, V3 and R1 using qRT-PCR. Mean values of expression data of wild-type and *spl-1* mutant plants were analyzed for statistical significance at *p* < 0.01 (**) level, as indicated by asterisks on top of bars.

To identify the candidate gene underlying the *lm1* locus of *spl-1* mutant, the expression patterns of all the eight candidate genes were tested in leaf tissues of wild-type and mutant parents at three different growth developmental stages, viz., V1, V3 and R1 [42], using qRT-PCR analysis. The oligo-nucleotides primers used for the qRT-PCR analysis are listed in Table S3. Out of these eight candidate genes, *Glyma.04g242300* showed significantly higher gene expression in wild-type relative to mutant genotype, and the expression was considerably lower in the *spl-1* mutant at all studied growth stages (Figure 8). The remaining seven candidate genes within the *lm1* locus revealed non-significant gene expression differences between wild-type and *spl-1* genotype at all stages. Therefore, the highly significant differential expression of *Glyma.04g242300* between wild-type and mutant genotype provided evidence for being the possible candidate genes responsible for leaf lesion mimic mutant phenotype of *spl-1* in soybean. To further clarify the sequence differences of the eight candidate genes, we sequenced the cDNA sequences of these genes. However, we did not find any nucleotide differences between the CDS sequences of *spl-1* and wild-type parents within the exons, and hence the difference might be in the 5′ or 3′ UTR region or non-coding region of this gene (the portion of gene not sequenced). Finally, depending on the above results, *Glyma.04g242300* is most likely to be involved in the lesion mimic phenotype of the *spl-1* mutant. However, it needs further functional validation to prove this function of *Glyma.04g242300*.

## 3. Discussion

### 3.1. spl-1 Is a New Soybean Leaf Lesion Mutant with Special Characteristics

To protect themselves against pathogen attack, plants have developed complicated defense mechanisms and signaling pathways. The HR results due to different pathways, and is a component of an effective defense system against biotrophic and hemibiotrophic pathogens. However, the molecular mechanisms and genes controlling the HR remain largely unknown [43]. In this regard, LMMs represent a broad group of phenotypes showing spontaneous cell death in the absence of disease pathogen, are interesting genetic materials for elucidating the molecular mechanism, pathways, and genes underlying HR and disease resistance. The appearance of lesions in different LMMs differs in induction conditions, timing, the extent of lesion spreading, color and size [44]. Thus far, some genes related to lesion mimic phenotype have been identified and cloned in different crop species, and their functions were also found diverse [2]. The results of these studies have indicated that lesion mimic phenotypes are regulated by different biological processes, thus hinting at the complexity of molecular mechanisms and signaling networks involved in HR and disease resistance [8].

Although a few LMMs have been characterized in soybean, the genetic mechanisms and pathways have not been well understood, and the genes regulating the mutant phenotype have not been cloned [14]. In this regard, the present study used a combined strategy of MutMap and traditional map-based cloning mapping to identify the candidate genomic region and genes underlying the LMM phenotype of *spl-1*. Chlorophyll a, chlorophyll b and total chlorophyll were significantly decreased in *spl-1* mutant relative to wild-type. Tetrapyrrole biosynthesis pathway leads to the production of chlorophyll a/b [45]. Hence, disruption of tetrapyrroles biosynthesis pathway at different stages leads to abnormal accumulation of photo-reactive molecules, which in turn leads to lesion-mimic phenotypes. For example, in the mutant *rugosa1* (*rug1)* tetrapyrroles biosynthesis pathway is affected at porphobilinogen deaminase (PGBD) that results in the accumulation of porphobilinogen [46]. Similarly, accumulation of protochlorophyllide (Pchlide) in *flu* and *oep16* mutants [47,48], uroporphyrinogen III in lesion 22 (*les22*) mutant [49] and coproporphyrinogen III in *lesion initiation 2/rice lesion initiation 1(lin2/rlin1)* mutants [50,51] leads to cell death phenotypes. Interestingly, cell death also results due to defects in chlorophyll catabolism. Indeed, disruption of two enzymes involved in the degradation of chlorophyll generates spontaneous lesions in the *accelerated cell death 1/lethal-leaf spot 1(acd1/lls1)* and *accelerated cell death 2 (acd2)* LMM [52,53,54]. Because of the known role of ROS during HR and in cells undergoing PCD, we further investigated the production of H_2_O_2_ as well as activities of antioxidants, viz., SOD, POD and CAT, and the content of lipid peroxidation (MDA) at different leaf position following the appearance of lesions in the lesion-mimic plants. Our study revealed that activities of all antioxidants, viz., SOD, POD, and CAT, were significantly increased in *spl-1* mutant in case of both HLM and LLM leaves except for CAT whose activity is reduced in LLM leaf of *spl-1* mutant compared to wild-type. This can be explained because lesions are present throughout the HLM leaf from bottom to top and are at final stage of development, whereas in the case of LLM leaf lesion mimic mutant phenotype is in its initial stage, i.e., yet to be developing, and lesions are very less in number usually at the bottom of leaf as well as small in size. Thus, it can be suggested that the CAT activity in leaves of *spl-1* depends upon the intensity and degree of lesion development.

Hence, the present study reported substantial accumulation of ROS, antioxidants and lipid peroxide in the leaves of LMM compared to wild-type, which is similar to the findings reported by Anand et al. [55], and thus suggested that mutation in *spl-1* mutant results in oxidative stress leading to PCD and brown necrotic lesions on the leaf surface [39].

### 3.2. Deploying MutMap and Traditional Mapping Methods to Accelerate Gene Identification

Conventional mapping is an important and effective strategy for identifying and isolating candidate genomic regions and genes for many crops. However, the general strategy for conventional mapping is time-consuming and laborious [23]. For example, *D53* gene encoding a protein that acted as a repressor of strigolactones in rice was identified by using 12,000 F_2_ plants [56]; Similarly, for the fine mapping of the recessive dialytic gene, *dl*, in tomatoes, 2248 F_2_ individuals were used [57]. In soybean, E1 a maturity locus gene involved in flowering time was delimited by using a very large number of individuals from F_2:3_ to F_2:5_ generations of soybean [58]. However, for the case of soybean, which is a larger crop plant requiring a huge area for sowing, it is impractical for growing so many progenies in the field. In this context, the last few decades have witnessed many reverse-genetic approaches that have become increasingly popular in some species, but map-based cloning is still an important approach for identifying candidate regions; however, BSA-seq methods facilitate and accelerate the gene identification process.

Therefore, in this study, we used an improved BSA-seq (MutMap) method that integrates the traditional BSA method with WGRS to rapidly identify specific genomic regions for the *spl-1* mutant phenotype of soybean. Moreover, the combination of MutMap and map-based cloning could effectively detect and fine map the QTL of interest. In the present study, major candidate genomic region underlying *spl-1* mutant phenotype was identified and mapped into a 76.23 kb genomic region on chromosome 04 by using an F_2_ and F_2:3_ mapping population via combined approaches of whole-genome NGS-based high-throughput MutMap and traditional mapping. MutMap analysis detected candidate genomic region, i.e., *lm1* locus based on ∆ (SNP-index) that were further validated and fine mapped by SSR traditional map-based cloning, and were mapped between the SSR markers BARCSOYSSR_04_1429 and BARCSOYSSR_04_1435, which suggests the validity and robustness of MutMap-seq as a strategy for quick and efficient scanning of major genomic region for mutant phenotype on a genome-wide scale in soybean. The merits of BSA-seq method relative to other traditional mapping approaches for identifying the major genomic regions governing plant height, seed weight, seedling vigor and flowering time in soybean, chickpea and rice have been recently reported [26,59,60]. MutMap takes advantage of the high-throughput WGRS and BSA. In addition, the use of an SNP-index provides accurate quantitative evaluation for the parental alleles’ frequencies, and also the genomic contribution from the two parents to F_2_ individuals. Hence, the above characteristics of MutMap make it a very efficient and faster approach for identifying genomic regions underlying mutant phenotype in soybean.

### 3.3. Candidate Genes for spl-1 Phenotype

In the present study, the major genomic region (*lm1* locus) governing mutant phenotype were delimited in a 76.23 kb physical interval on chromosome 04 by using the combined strategy of MutMap and traditional map-based cloning analysis. Eight genes were predicated within this region, and the functional annotations of six genes are known, whereas the annotation of the remaining two genes was not available (Table 3). Based on the functional annotation and qRT-PCR expression analysis *Glyma.04g242300* was suggested to be a possible candidate gene for governing lesion mimic phenotype of *spl-1* mutant. The *Glyma.04g242300* is a family protein gene in *Arabidopsis* (At2G02850) that belongs to plantacyanin (PLC), which is a plant-specific phytocyanin (PC) sub-family of blue copper proteins functioning in the electron transport chain of photosynthesis [41,61]. It serves as an electron transfer agent in the cytochrome complex which follows Photosystem II and the entry point to Photosystem I of the non-cyclic electron transfer process. Defects in photosynthetic electron transport will affect photosynthesis process as well as chlorophyll catabolism and cell death [62]. Recently, it has been revealed that *OsUCL8* (*Oryza sativa* Uclacyanin like protein 8), a rice plantacyanin gene could regulate grain yield and photosynthesis [63]. *OsUCL8* cleaved by *miR408* affects copper homeostasis in the plant cell, which in turn affects the abundance of plastocyanin proteins and photosynthesis [63]. In the present study, soybean *spl-1* mutant revealed a significant reduction of chlorophyll content compared to wild-type, which indicates that the degradation of chlorophyll generates spontaneous chlorotic leaves. Previous studies have indicated that PCs are involved in various plant activities, including cell differentiation and reorganization [64], pollen tube germinating and anther pollination [41,65]. Hence, these studies indicate that the PC gene family can have multiple functions during plant development. Several researchers have indicated that salt and drought stresses can induce the expression of PC genes, suggesting the potential response to abiotic stresses [66,67]. Microarray data also suggest that plantacyanins may be stress-related proteins and be involved in plant defense responses [68,69]. It is assumed that plantacyanin is one of the targets of microRNAs that regulates transcription factors involved in different aspects of plant development [70,71], and miR408 regulates photosynthesis via plantacyanin [63,72]. Therefore, it is reasonable to postulate that *Glyma.04g242300* is the candidate gene for lesion mimic mutant phenotype of *spl-1* in soybean. However, further evidence is needed to functionally validate this hypothesis.

Hence, the results of the present study provide new gene resources in soybean that might regulate the leaf LMM phenotype of *spl-1* mutant. This increases our current knowledge of genes involved in PCD and HR in soybean. By analyzing the function, these genes will help to elucidate the mechanism as well as pathways involved in the development of lesion mimic phenotype in soybean. This in turn will provide explanation how plants regulate PCD as well as develop HR for resistance against biotic stresses, and hence will greatly help to develop disease-resistant soybean varieties to overcome the losses that occur due to disease constraint.

## 4. Materials and Methods

### 4.1. Plant Materials and Phenotypic Evaluation

Plant material of the present study included soybean accessions, viz., NN 1138-2, Williams 82 (W82) and PI378692, which were obtained from National Center for Soybean Improvement, Nanjing Agricultural University, Nanjing, Jiangsu Province, China. The leaf lesion mimic mutant (LMM) called spotted leaf-1 (*spl-1*) was identified from the EMS-induced mutational library of the cultivar “NN 1138-2” that was treated with 0.5% (*w*/*v*) EMS for 12 h. The M_1_ seeds were harvested and pooled together. Subsequently, M_2_ plants were individually harvested. Furthermore, M_2:3_ lines showed segregation for mutant and normal phenotype at V1 and V2 stages [42]. Seeds from 10 individual plants that had normal leaves were harvested and sown for M_4_ generation. Progeny obtained from the normal heterozygous M_2:3_ lines also exhibited segregation of normal and lesion mimic phenotypes. The same selection and planting procedures were conducted through M_4_ to M_10_ generations. In each generation, some lines showed segregation of the normal and disease-like leaf phenotypes, with a 3:1 ratio, indicating that a single recessive allele might control the disease-like leaf trait. Through consecutive selfing and selection, we achieved M_9:10_ lines, and the mutant plants that have 99.8% homogeneity to the normal M_9:10_ plants were bulked together. These mutant lines were named as *spl-1* (spotted leaf-1) and were obtained from heterozygous individuals.

Seeds of mutant *spl-1*, W82, and PI378692 were planted at Jiangpu Agricultural Experiment Station, Nanjing Agricultural University in 2015, and two crosses were made at flowering time, viz., W82×*spl-1* and PI378692×*spl-1*. Mutant parent (*spl-1*) was used as a male parent in both crosses. The F_1_ seeds obtained from each cross were planted at Jiangpu Station in next year cropping season, i.e., 2016, and no F_1_ plants showed mutant phenotype, indicating recessive nature of the mutant trait. F_2_ seeds derived from F_1_ plant were harvested separately from both crosses. The F_2_ population and F_2:3_ families of each cross along with their parents were grown in the cropping seasons of 2017 and 2018, respectively, at the Jiangpu Station. Phenotypic data (normal and lesion mimic) of parents, F_1_, F_2_, and F_2:3_ plants were recorded at V1–V5 and R1 growth stages of soybean under normal field conditions [42]. These F_2_ and F_2:3_ populations derived from W82×*spl-1* and PI378692×*spl-1* were used for mapping of the mutant locus. Chi-square analysis was applied to test the goodness-of-fit of observed to the expected ratio for independent assortment or linkage in all populations.

### 4.2. Leaf Pigment Quantification and Histological Analyses

Leaf photosynthetic pigments were extracted from leaves at seedling and mature stages collected from the same position/rank of both wild-type and mutant plants [42]. Fresh leaf sample of 0.1 g was taken and cut into small pieces, then steeped in 80% acetone at room temperature for 24 h. The quantification of pigments was performed using a Tecan Infinite Pro Microplate Reader (Tecan Austria GmbH, Grodig, Austria) following the method reported previously [73]. Pigments measurement was conducted for three independent experiment repeats. All these experiment operations were carried out in the dark to avoid degradation of photosynthetic pigments.

For histological analysis, third top leaves of both mutant and the wild-type plants were collected from the 35-day-old plants when lesions mimic phenotypes fully appeared. The 10 µm leaf sections of both wild-type and mutant were obtained using ultra-microtome (Leica EM UC7, Leica Microsystems Inc., Buffalo Grove, IL, USA) with three replicates. Leaf sections were prepared for histological analysis following the method of Carland and McHale [74] with some modification, and further leaf sections were stained with 0.1% safranine. Images were observed with an optical microscope under different magnification (Zeiss Axioplan, Jena, Germany), and were captured by a digital camera connected with the microscope. Parameters, viz., central meta-xylem shape, number of xylem and phloem vessels, spongy and palisade mesophyll parenchyma, were observed and recorded for comparative analysis.

### 4.3. Leaf Histochemical and Physiological Analyses

#### 4.3.1. Trypan Blue Staining of Cell Death

Leaf samples of both the mutant and wild-type plants were collected at the same position/rank for Trypan blue staining when mutant phenotype appeared on the leaves of mutant plants at mature developmental stage. Leaves were stained with Trypan blue (Sigma-Aldrich, St. Louis, MO, USA) according to the method previously described by Chen et al. [75], with some modifications. Briefly, plant tissues were submerged in a 70 °C Trypan blue solution (2.5 mg of Trypan blue per mL, 25% (wt/vol) lactic acid, 23% water-saturated phenol, 25% glycerol, and H_2_O) for 10 min, and then heated over boiling water for 2 min and left to stain overnight. Stained leaves were washed several times with absolute ethanol to remove Trypan blue solution until the leaves become colorless. Finally, ethanol was discarded, and the leaf samples were covered with 70% glycerol for visualization of cell death under microscopic analysis. The staining procedure was done in triplicate (three times).

#### 4.3.2. H_2_O_2_ Detection by DAB

Hydrogen peroxide (H_2_O_2_) was detected by submerging the leaf samples of both wild-type and *spl-1* mutant in a 3,3′-diaminobenzidine (DAB) solution according to Rahman et al. [76], with some modifications. Briefly, leaves from wild-type and mutant plants were taken for DAB staining after lesions appeared (30 days and 60 days after sowing), and incubated in a 0.1% (*w*/*v*) DAB (10 mM MES, pH 7.0) solution at 25 °C temperature in the dark with gentle shaking for 12 h or more depending upon visibility of spots. Leaves were thoroughly washed in ddH_2_O several times until DAB solutions were completely removed. Then, the chlorophyll was cleared by treating with 95% (*v*/*v*) ethanol boiling for 10 min. The transparent leaves were observed and photographed in 70% glycerol.

#### 4.3.3. Antioxidants Activities and Lipid Peroxidation Determination

Activities of antioxidants, viz., Superoxide dismutase (SOD), Peroxidase (POD) and Catalase (CAT), as well as the content of lipid peroxidation (MDA), were determined for both the wild-type and mutant plants. High-lesion mimic (HLM) and low-lesion mimic (LLM) leaves were collected at the third and second position from the top of the same mutant plant, respectively, and were compared with the corresponding leaves of wild-type plants from the same position. In the case of HLM leaves, the lesion mimic mutant phenotype was well developed, and lesions were present throughout the leaf surface, whereas for LLM leaves, lesion were yet to be developed, were much fewer in number, and were not present throughout the leaf, usually on the bottom of leaf. The activities of SOD, POD and CAT as well as MDA content of the leaves were determined using SOD Assay Kit (T-SOD, A001-1), POD Assay Kit (A084-3), CAT Assay Kit (A007-1) and MDA Assay Kit (A003) by following the manufacturer’s protocol (Nanjing Jiancheng Bioengineering Institute, Nanjing, China) and Li et al. [77]. Briefly, fresh leaf samples (1.0 g) of six-week-old plants were sliced and homogenized in mortar and pestle with 9 mL ice-cool 10× phosphate buffered saline (PBS) (pH 7.2–7.4) (Beijing Solarbio Science & Technology Co., Ltd., Beijing, China). The homogenates were further centrifuged at 3500 rpm for 10 min at 4 °C, and the supernatants were collected and used as crude extracts for above-cited assays by using a UV-1800 Shimadzu, spectrophotometer (SHIMADZU Corporation, Kyoto, Japan). Three independent samples were assayed, and standard errors (SE) among them were calculated.

### 4.4. MutMap Analysis

#### 4.4.1. Construction of MutMap Libraries and Illumina Sequencing

Genomic DNA was isolated from young leaves of soybean using DNAquick Plant System (TIANGEN Biotech, Beijing, China) according to the manufacturer’s protocol. DNA samples were quantified using Qubit^®^ 2.0 Fluorometer (Thermo Scientific, Waltham, MA, USA). Two DNA bulks/pools, viz., wild-type pool (Pool A), and mutant pool (Pool B), were generated for Illumina libraries by pooling equal amounts of DNA from 20 wild-type and 20 mutant F_2_ genotypes of W82×*spl-1* cross. About 5–10 µg of DNA from two pools were used to construct paired-end sequencing libraries, which were sequenced with an IlluminaHiSeq^®^ 2500 (Illumina Inc., San Diego, CA, USA) NGS platform. FASTQ raw sequence reads with a minimum phred Q-score of 30 across >95% of nucleotide sequences were considered as high quality. The quality of these sequences was further checked by using FASTQC v0.10.1 (Babraham Institute, Cambridge, UK). High-quality FASTQ filtered sequences obtained from two DNA pools were aligned and mapped to the *Glycine max* Wm82.a1.v1 reference genome from Phytozome [40] using Burrows–Wheeler alignment tool (BWA v0.7.10, Cambridge, UK) with default parameters [78]. High-quality SNPs (minimum sequence read depth: 7 with SNP base quality ≥20) were discovered using SAM tools (Cambridge, UK) [79] by following the detailed procedure of Takagi et al. [80] and Lu et al. [31].

#### 4.4.2. SNP Index Analysis

SNP-index was calculated at each SNP position for both Pool A and Pool B, which represented the parental alleles of the population [81,82]. A ∆ (SNP-index) was calculated by subtraction of the Pool A SNP index from the Pool B SNP index [26,80,83,84]. Hence, the SNP locus with high ∆ (SNP-index) value is an indicator an allele was highly common in Pool A and depleted in Pool B. If there is no major candidate region/locus of the target gene in a genomic region, the Δ (SNP-index) value should not be significantly different from 0. Using a null hypothesis of no QTLs, 95% confidence intervals of the Δ (SNP-index) for all the SNP positions were calculated with given read depths and plotted these against the Δ (SNP-index) [80].

#### 4.4.3. Sliding-Window Analysis

In a given genomic interval, the average distributions of the SNP-index and ∆ (SNP-index) were estimated by using sliding window approach with a 2-Mb window size and 50-kb sliding step, and these data were used to plot SNP-index plots for all soybean chromosomes. Genomic regions that showed average ∆ (SNP-index) significantly higher than surrounding region and windows revealed an average *p*-value < 0.05 were considered candidate genomic regions harboring a locus associated with the mutant phenotype of *spl-1* soybean mutant [80].

### 4.5. Fine Mapping of lm1 Locus

To verify the accuracy of the *lm1* genomic region identified by MutMap analysis and establish mapping reliability of this approach, a traditional map-based cloning genetic linkage method was performed to find out the linkage of molecular markers and phenotypic loci of *spl-1.* The two F_2_ populations, viz., W82×*spl-1* and PI378692×*spl*-*1*, were used for this purpose, and a total of 130 SSR markers in the predicted region on chromosome 04 were selected to survey the polymorphism between the wild-type and *spl-1* mutant lines [85]. Polymorphic markers that may be linked with the mutated genes were screened using the method of bulked-segregant analysis (BSA), as proposed by Michelmore et al. [86]. Both wild-type and mutant groups contained 10 randomly selected F_2_ individuals, and the protocol was according to Wang et al. [19]. Within each group, the DNA from all individuals was pooled using an equal amount of DNA from each plant. The mapping steps involve as: the polymorphic SSR markers between two parents of cross populations were identified; then, the pools of wild-type and mutant plants were screened with those SSR markers that were polymorphic between the parents to identify markers for screening the F_2_ populations. Mapmaker 3.0 software (Whitehead Institute for Biomedical Research, Cambridge, MA, USA) was used to identify the linkage between SSR markers and target genes [87]. A total of 307 recessive mutant plants from the F_2_ and F_2:3_ populations of W82×*spl-1* and PI378692×*spl-1* crosses were used for preliminary and fine mapping (Table 1), and the protocol was according to Wang et al. [19].

For the PCR amplification of marker genotyping, 10 ng DNA was used in 10 μL system under the instruction of the Taq Master Mix (Novoprotein, Shanghai, China). PCR thermal cycler was programmed as follows: initial denaturation at 95 °C for 5 min; followed by 32 cycles of denaturation at 95 °C for 30 s, annealing at 55 °C for 40 s and extension at 72 °C for 50 s; with final incubation at 72 °C for 10 min before hold to 4 °C. The amplification product was separated on 8% non-denaturing polyacrylamide gels that were stained with 1 g·L^−1^ AgNO_3_ for 15 min before visualizing with 16 g·L^−1^ NaOH plus 11 mL·L^−1^ CH_3_OH for 10 min.

### 4.6. Expression and Sequence Analysis of Candidate Genes

To analyze the candidate genes underlying the *lm1* locus of *spl-1* mutant, we investigated the expression pattern of all the eight genes present within *lm1* locus using real-time quantitative PCR (qRT-PCR). Young leaf samples at three different growth stages, viz., V1, V3, and R1, were collected from the wild-type and mutant parents. Total RNAs from the leaves were isolated by using the RNA prepare plant kit (TIANGEN, Beijing, China). First-strand cDNA was synthesized using two-step PrimerScript^TM^ RT reagent Kit gDNA Eraser (TaKaRa, Kusatsu, Shiga, Japan) according to the manufacturer’s instructions. Real-time qRT-PCR was performed using a ChamQ SYBR qPCR Master Mix (Vazyme, Jiangsu, Nanjing, China) on a Bio-Rad system. Primers were designed by Beacon Designer 7.9 software (Premier Biosoft International, Palo Alto, CA, USA). *GmActin11* was used as an internal control for the qRT-PCR analysis, and three biological replicates were used for each reaction. Average relative expression levels for wild-type and mutant parent were calculated. One-way ANOVA tests were performed by IBM-SPSS software to test the significance of differences in expression levels among different samples.

For further verification, we sequenced the coding sequence (CDS) of eight candidate genes, viz., *Glyma.04g242300*, *Glyma.04g242400*, *Glyma.04g242500*, *Glyma.04g242600*, *Glyma.04g242700*, *Glyma.04g242800*, *Glyma.04g242900* and *Glyma.04g243000*, in the wild-type and mutant parents for the identification of nucleotide differences and possible candidate gene responsible for lesion mimic mutant phenotype of *spl-1*. The homologous localized region and sequences of candidate genes were obtained from the database of Phytozome (https://phytozome.jgi.doe.gov), and SoyBase (http://soybase.org/). RNA was isolated according to the above-mentioned protocol. Transcript two-step gDNA Removal was used for reverse transcription, and Prime Script™ RT Reagent Kit (TaKaRa, Kusatsu, Shiga, Japan) was used for cDNA synthesis. Primers for qRT-PCR were designed by Primer Premier 5.0 software (Premier Biosoft International). The target gene was subjected to PCR by using Phanta^®^ Max SuperFidelity DNA Polymerase from Vazyme, and sent to GeneScript^®^, China for sequencing. The alignments of the nucleotide sequences were performed using BioXM software.

## 5. Conclusions

In this study, we isolated a new soybean leaf lesion mimic (*spl-1*) mutant, in which necrotic lesions started to first visualize on the aged/older leaves, and, finally, the whole leaf became chlorotic yellow. The *lm1* locus controlling mutant phenotype of *spl-1* was fine-mapped in a 76.23 kb genomic region harboring eight candidate genes, and among them, *Glyma.04g242300* was considered to be the possible candidate gene for the mutant phenotype of *spl-1*. We speculate that mutation in this gene affected chlorophyll degradation, resulted in oxidative stress and increased antioxidant activities, which in turn led to necrotic lesions and PCD, and we also suggest this gene may be related for resistance to disease and stress. However, further studies are required for detailed investigation of the actual molecular mechanism and signaling pathways involved in the PCD. The results obtained in this study provide a foundation for the cloning and validating the *lm1* gene of *spl-1* mutant.

## Figures and Tables

**Table 1:** Chi-square test for segregation ratio of normal and mutant plants in the F_2_ and F_2:3_ lines in two crosses viz., W82×*spl-1* and PI378692×*spl-1*.

Cross.	Generation	No. of Plants/Lines				Expected Ratio	χ^2^	*p*
		Total	Wild Type	Segregation	Mutant			
W82×*spl-1*	F_2_	400	310	-	90	3:1	1.20	0.27
	F_2:3_ line	20	6	14	0	1:2	0.01	0.94
PI378692×*spl-1*	F_2_	793	609	-	184	3:1	1.27	0.26
	F_2:3_ line	13	4	9	0	1:2	0.01	0.92

**Table 2:** Polymorphic simple sequence repeat (SSR) markers used to narrow down the *lm1* locus.

Marker	Chromosome	Start *	End	Primer (F/R) Sequences	
BARCSOYSSR_04_1390	Gm04	50549768	50549787	F	CCCGGTACAGTTGAGATGGA
		50550014	50549995	R	TTGCACTTCAGTAGGCCCTC
BARCSOYSSR_04_1391	Gm04	50588500	50588519	F	AGATGGTGGTGTTCTCAGGG
		50588766	50588747	R	ACCATCACCAACATGCAGAT
BARCSOYSSR_04_1418	Gm04	50942037	50942061	F	TTTTTCTTCAGAAACTTGAAACATT
		50942254	50942234	R	TGCATTTCTGAAACAAGGCAT
BARCSOYSSR_04_1420	Gm04	50953149	50953174	F	AAGTGATCAATGTTATCGATGAAGTA
		50953433	50953409	R	TTTGTCTCAATTAGTGTGGAATTTG
BARCSOYSSR_04_1426	Gm04	51011052	51011071	F	ATCAGAGGTCTGCCACCAAT
		51011271	51011252	R	CGCTGACAGACACCAAGAGA
BARCSOYSSR_04_1429	Gm04	51035485	51035504	F	TTTGCTACAGTGCTATCGGC
		51035766	51035747	R	TGCCAGCCGCTTATCTATCT
BARCSOYSSR_04_1435	Gm04	51111716	51111735	F	GTCCGTGCCAGTTTTTCATT
		51111960	51111941	R	TGCTGCACTTTCTCCTGATG

* Location has taken from Glyma2.0; F (forward primer), R (reverse primer).

**Table 3:** Functional annotation of eight candidate genes located within the *lm1* locus/region identified through fine mapping.

Gene ID	Position (bp)	Direction	Function Annotation
Glyma.04g242300	51036742-51037897	Forward	Plantacyanin
Glyma.04g242400	51047485-51048880	Forward	Unknown
Glyma.04g242500	51053346-51056055	Reverse	Flavin-binding monooxygenase family protein
Glyma.04g242600	51061920-51064096	Forward	Unknown
Glyma.04g242700	51064744-51067380	Reverse	F-box/RNI-like superfamily protein
Glyma.04g242800	51082744-51092913	Reverse	ACT domain repeat 3
Glyma.04g242900	51103163-51108385	Reverse	Protein kinase superfamily protein
Glyma.04g243000	51109534-51115501	Reverse	Thiamin diphosphate-binding fold (THDP-binding) superfamily protein

## Supplementary Materials

Supplementary Materials can be found at https://www.mdpi.com/1422-0067/20/9/2193/s1.

## Abbreviations

LMMs	lesion mimic mutants
*spl-1*	spotted Leaf-1
*lm1*	lesion mimic 1
PCD	programmed cell death
HR	hypersensitive response
ROS	reactive oxygen species
SOD	superoxide dismutase
POD	peroxidase
CAT	catalase
MDA	malondialdehyde
SSR	simple sequence repeat
PLC	plantacyanin
PC	phytocyanin
qRT-PCR	quantitative real-time PCR
NGS	next-generation sequencing
WGRS	whole genome re-sequencing
DAB	3,3′-diaminobenzidine
SNP	Single Nucleotide Polymorphisms
