# Supplementary material for: Characterization and Rapid Gene-Mapping of Leaf Lesion Mimic Phenotype of spl-1 Mutant in Soybean (Glycine max (L.) Merr.)
Source: Int J Mol Sci. 2019 May 3;20(9):2193. doi: 10.3390/ijms20092193 (PMC6539437; doi:10.3390/ijms20092193)
Supplement: Supplementary file 1 [file ijms-20-02193-s001.zip › Supplementary Figure S1.docx]

**Supplementary Figure S1:** SNP-index of wild-type (A-Pool) and *spl-1* mutant (B-Pool) pool, and their combined Δ (SNP-index) graph. X-axis indicate physical position of chromosome and Y-axis indicate the average SNP-index in 2 Mb interval with a 50 kb sliding window. (A), (B) & (C) Figures represent SNP-index of wild-type and *spl-1* mutant and their combined Δ (SNP-index), respectively.


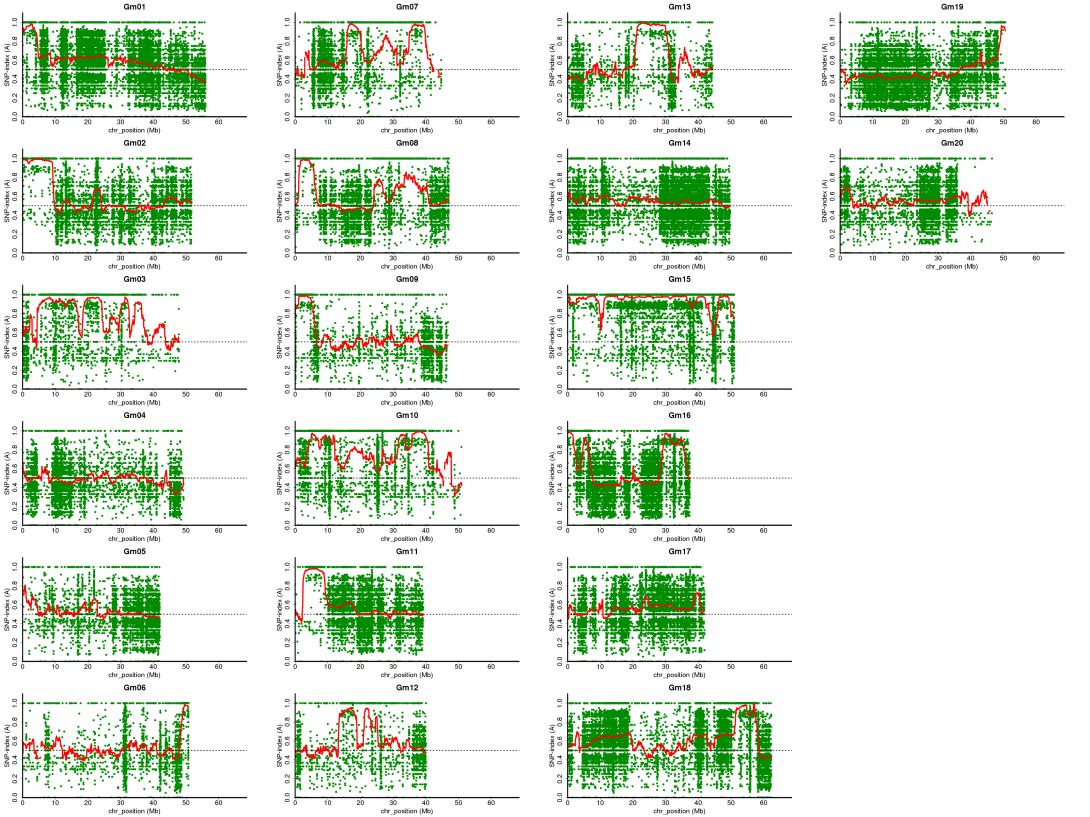
**(A)**

(B)
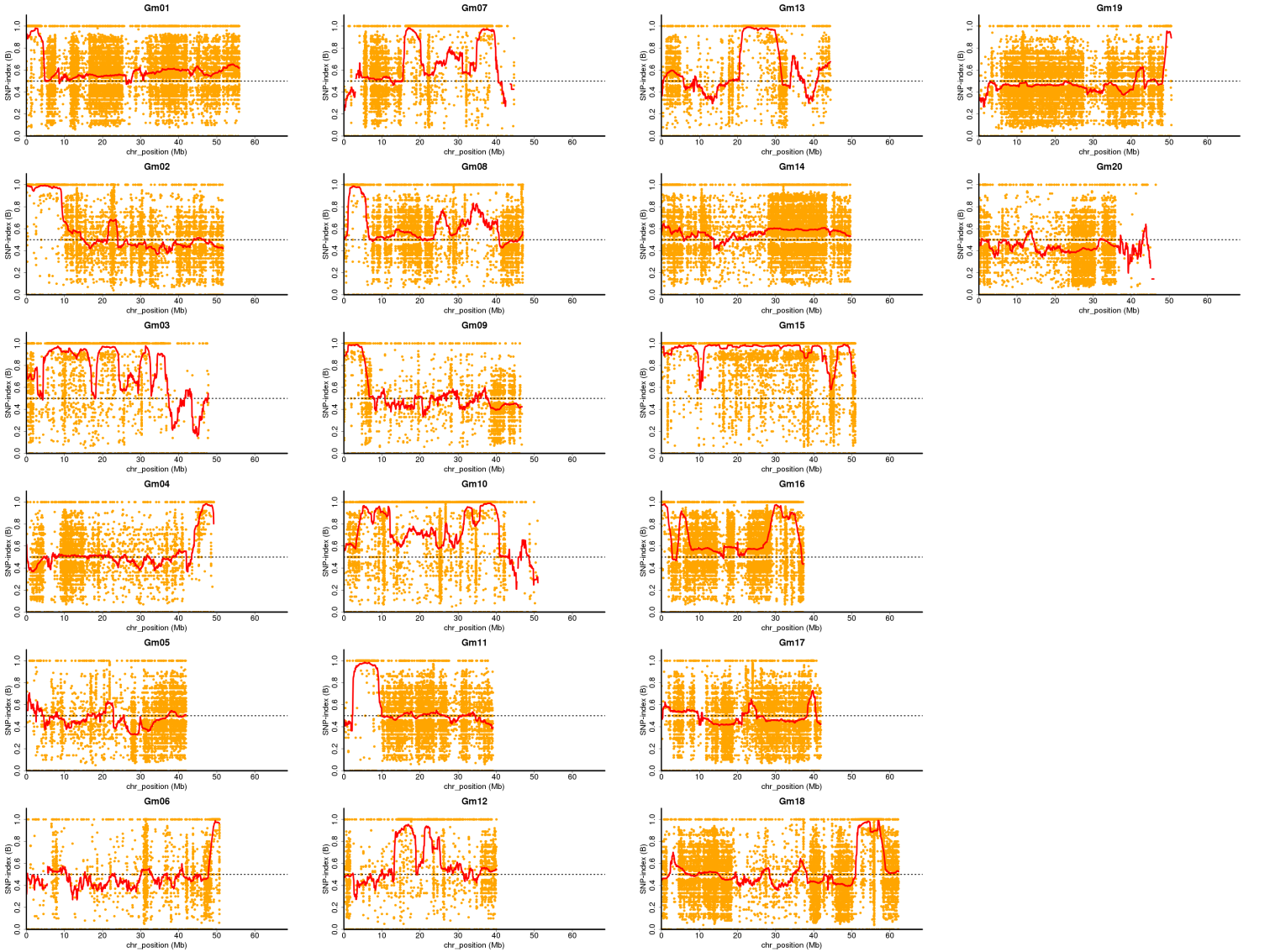


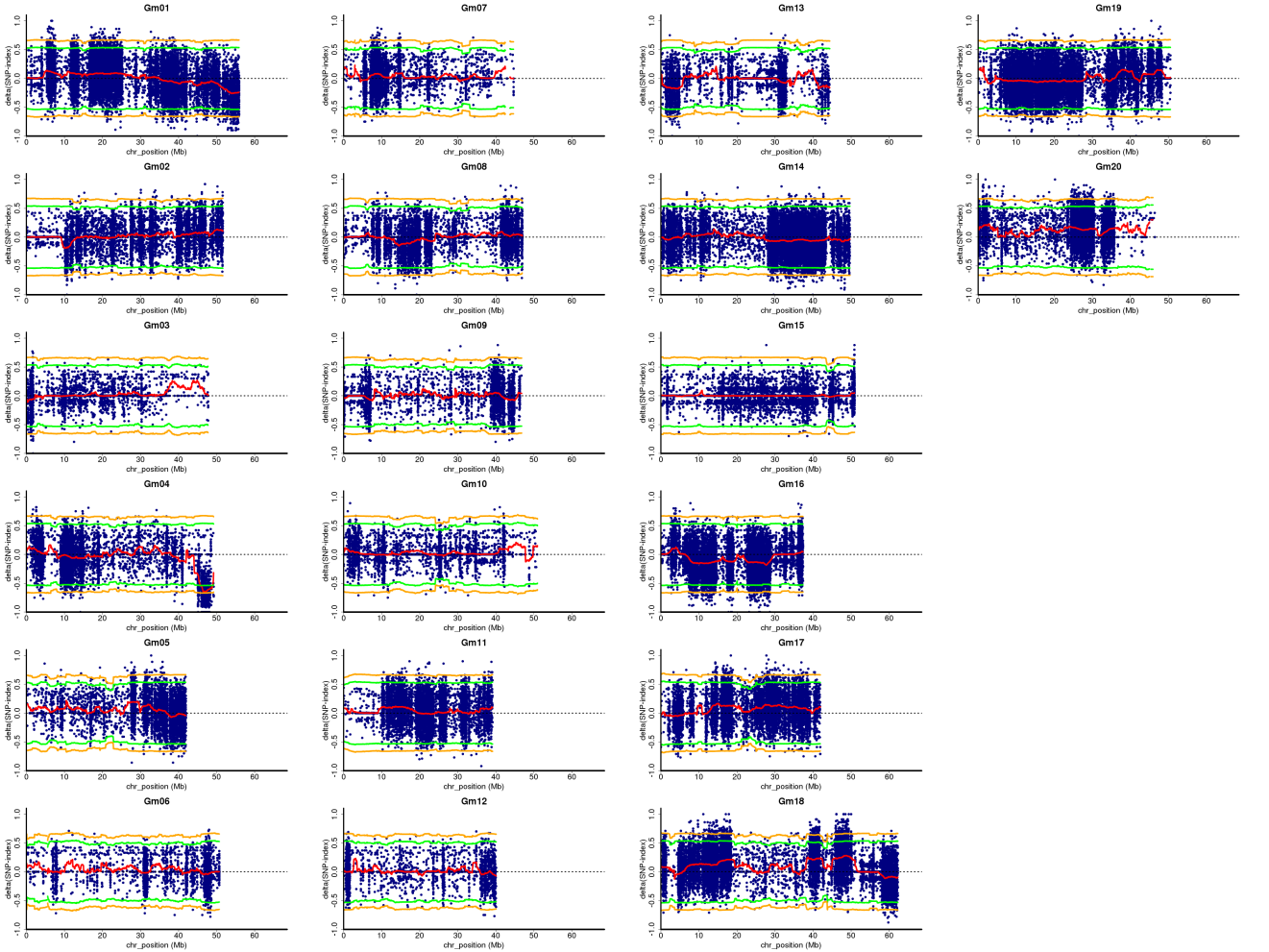
(C)
